# Geraniin Protects against Cerebral Ischemia/Reperfusion Injury by Suppressing Oxidative Stress and Neuronal Apoptosis via Regulation of the Nrf2/HO-1 Pathway

**DOI:** 10.1155/2022/2152746

**Published:** 2022-02-18

**Authors:** Yuan Yang, Bo He, Xiaochao Zhang, Renhua Yang, Xin Xia, Lu Chen, Rui Li, Zhiqiang Shen, Peng Chen

**Affiliations:** School of Pharmaceutical Sciences and Yunnan Key Laboratory of Pharmacology for Natural Products, Kunming Medical University, Kunming 650500, China

## Abstract

Geraniin, a polyphenol isolated from *Phyllanthus amarus*, possesses extensive biological and pharmaceutical activities. In this study, we investigated the protective effect against cerebral ischemia/reperfusion (I/R) injury of geraniin and explored its potential mechanism. Middle cerebral artery occlusion/reperfusion (MCAO/R) was used to simulate cerebral I/R injury in vivo, and oxygen-glucose deprivation/reoxygenation (OGD/R) was applied to establish an in vitro model of cerebral I/R injury. In this study, we performed TTC and HE staining and adopted a neurological score method to evaluate the neuroprotective effect of geraniin in vivo and used the CCK-8 assay to assess this effect in vitro. Indices of reactive oxidation capacity were measured in vivo and in vitro to verify the antioxidant capacity of geraniin. TUNEL staining and flow cytometry were applied to measure the apoptosis rate, and Western blotting was performed to assess the expression of apoptosis-related proteins. Finally, the expression of Nrf2 and HO-1 was evaluated in vivo and in vitro by Western blotting. Geraniin significantly reduced the infarct volume, decreased neurological deficit scores, alleviated pathological changes in neurons, and increased the cell survival rate. Geraniin increased the activity of superoxide dismutase (SOD) and decreased the activity of lactate dehydrogenase (LDH) and the contents of malondialdehyde (MDA), nitric oxide (NO), and neuronal nitric oxide synthase (nNOS) in vivo and in vitro. In addition, geraniin significantly reduced the apoptosis. Furthermore, geraniin also evidently increased Nrf2 (total and nuclear) and HO-1 protein expression in vivo and in vitro. Collectively, these results imply that geraniin may exert a protective effect against cerebral I/R injury by suppressing oxidative stress and neuronal apoptosis. The mechanism underlying the protective effect of geraniin is associated with activation of the Nrf2/HO-1 pathway. Our results indicate that geraniin may be a potential drug candidate for the treatment of ischemic stroke.

## 1. Introduction

Stroke, a common cerebrovascular disease in the clinic, can be divided into ischemic and hemorrhagic stroke and remains the predominant cause of death and disability in both developed and developing countries [1]. Ischemic stroke, which is caused by sudden interruption of blood flow to the brain, resulting in brain cell death and neurological impairment, is the most prevalent type of stroke and accounts for approximately 75% of all stroke cases [2]. Currently, early restoration of blood supply after ischemic stroke is considered the main treatment strategy for improving clinical outcomes. However, the reperfusion processes may further exacerbate the initial ischemic injury after ischemic attack, and this process is termed cerebral ischemia/reperfusion (I/R) injury [3]. Although much progress has been made in the study of the mechanism underlying cerebral I/R injury in recent years, the pathogenesis of the disease is still not fully elucidated, and there is a lack of effective treatments [4].

Oxidative stress is one of the most important processes in cerebral I/R injury and is also a major risk factor for cell apoptosis [5]. Studies have shown that oxidative stress is elevated in rat brain tissue after middle cerebral artery occlusion/reperfusion- (MCAO/R-) induced injury, as the activity of superoxide dismutase (SOD) is decreased, the activity of lactate dehydrogenase (LDH) is increased, and the contents of oxidative stress markers such as malondialdehyde (MDA), nitric oxide (NO), and neuronal nitric oxide synthase (nNOS) are increased [6]. Moreover, excessive accumulation of free radicals can lead to damage to macromolecules such as proteins and DNA and lipid peroxidation and accelerate neuronal apoptosis during the development of cerebral I/R injury [7]. Oxidative stress is closely associated with cell apoptosis, which can trigger the cell death pathway involving Bcl-2 family proteins and subsequently contribute to activation of the caspase cascade and proteolysis [8]. Sun and Cui found that cerebral I/R injury significantly reduces the activity of the antioxidant enzyme SOD, increases the content of prooxidant factors such as MDA, and exerts proapoptotic effects by upregulating Bax and caspase-3 expression and downregulating Bcl-2 expression [9]. These studies indicate that oxidative stress and cell apoptosis play important roles in the pathogenesis of cerebral I/R injury. Therefore, antioxidant and antiapoptotic agents have been the focus of studies on the use of neuroprotective drugs for the prevention and treatment of cerebral stroke.

Previous studies have revealed that decreased expression of nuclear factor E2-related factor 2 (Nrf2) and heme oxygenase-1 (HO-1) contributes to aggravation of brain damage by increasing the infarct volume and decreasing neurological function, during cerebral ischemia [10]. Many previous studies have shown that natural products or Chinese herbal medicines exert neuroprotective effects against cerebral ischemia by activating the Nrf2/HO-1 pathway. Fu et al. found that pelargonidin can effectively reduce the infarct volume and improve neurological function in rats subjected to middle cerebral artery occlusion (MCAO), thereby enhancing memory and learning, and that the potential mechanism is associated with activation of the Nrf2/HO-1 signaling pathway [11]. Thus, the Nrf2/HO-1 signaling pathway is a key protective pathway against oxidative stress and apoptosis.

Geraniin is a polyphenol isolated from the medicinal plant *Phyllanthus amarus.* Geraniin has antioxidative, anti-inflammatory, antithrombotic, and other biological activities. Studies have reported that geraniin protects bone marrow-derived mesenchymal stem cells from H_2_O_2_-induced oxidative stress injury via the PI3K/Akt pathway [12]. Another study revealed that geraniin mitigates lipopolysaccharide- (LPS-) elicited neural/synaptic neurodegeneration, amyloidogenesis, neuroinflammation, and cognitive impairment and suggested geraniin as a therapeutic option for neuroinflammation-associated neurological disorders such as Alzheimer's disease [13]. In addition, we performed some studies on geraniin and found that geraniin can inhibit platelet aggregation, suppress platelet-neutrophil interactions, and exert an antithrombotic effect [14]. Moreover, our previous studies indicated that geraniin has antiosteoporotic effects, promoting the osteogenic differentiation of osteoporotic large bone marrow mesenchymal stem cells in vitro and increasing the expression of Wnt3a and *β*-catenin [15]. Although there have been many reports on the pharmacological effects of geraniin, research on the neuroprotective effects of geraniin and its molecular mechanism is limited.

In this study, we adopted a rat model of MCAO/R and a PC12 cell model of oxygen-glucose deprivation/reperfusion- (OGD/R-) induced oxidative stress injury to investigate the protective effect of geraniin and its potential molecular mechanism of action. This study provides a theoretical and experimental basis for geraniin as a new therapeutic drug for stroke and oxidative stress-related diseases.

## 2. Materials and Methods

### 2.1. Chemical Reagents

Geraniin (purity > 99%) was kindly provided by Professor Jikai Liu (Kunming Institute of Botany, Chinese Academy of Science, Kunming, China). Nimodipine injection was obtained from Bayer Pharmaceutical Co., Ltd. (Guangzhou, China). 2,3,5-Triphenyltetrazolium chloride (TTC) was purchased from Solarbio Science & Technology Co., Ltd. (Beijing, China). SOD and MDA, LDH, NO, and nNOS kits were obtained from Nanjing Jiancheng Bioengineering Institute (Nanjing, China). A TdT In Situ Apoptosis Detection Kit was purchased from R&D Systems, Inc. (Minneapolis, USA). An Annexin V-FITC Apoptosis Detection Kit was obtained from KeyGEN Biotech Co., Ltd. (Nanjing, China). Dulbecco's modified Eagle's medium (DMEM), fetal bovine serum (FBS), and penicillin-streptomycin were obtained from Gibco (Grand Island, NY, USA). All other chemicals and solvents used were of either analytical grade or pharmaceutical grade.

### 2.2. Animal Experiments and Ethics Statement

Male specific pathogen-free- (SPF-) grade Sprague-Dawley (SD) rats weighing 280–320 g were provided by the Laboratory Animal Center of Kunming Medical University (license number: SCXK (Yunnan) k2020-0006). The animals were housed on a 12/12 h light/dark cycle at room temperature (24 ± 1°C). The rats were provided free access to rodent diet and tap water and subjected to adaptive feeding for one week. All procedures were conducted in strict accordance with the Chinese Legislation on the Use and Care of Laboratory Animals and approved by the Medical Ethical Committee of Kunming Medical University. The MCAO/R model was established as previously described by our group [16]. Briefly, after anesthetization with 2% isoflurane, the rats were fixed to an operating plate in the supine position, and the hair above the cervical spine was carefully removed with an electric razor. Then, the right common carotid artery (CCA) and external carotid artery (ECA) were isolated and ligated. A monofilament nylon suture with a rounded tip (diameter: 0.47 mm) was gently inserted into the internal carotid artery (ICA) through the ECA to occlude the origin of the middle cerebral artery. During the operation, the rats were placed on a heating pad, and the thread was removed after 2 h of ischemia to allow reperfusion for 72 h.

### 2.3. Experimental Groups

All rats were randomly assigned to the following six groups (*n* = 8 each): (1) the sham-operated (sham) group, (2) the MCAO/R group, (3) the 5 mg/kg geraniin-treated group, (4) the 10 mg/kg geraniin-treated group, (5) the 20 mg/kg geraniin-treated group, and (6) the 1 mg/kg nimodipine-treated group. Geraniin was dissolved in dimethyl sulfoxide (DMSO) and diluted to different concentrations with normal saline. The sham-operated rats underwent the same protocol but without MCA ligation. Two hours after the cerebral ischemia, geraniin (5, 10, and 20 mg/kg·d) and nimodipine (1 mg/kg·d) were intraperitoneally injected into the rats, and the agents were administered continuously for three days.

### 2.4. Neurological Deficit Evaluation

After reperfusion for 72 h, neurological deficits were assessed according to the following scoring criteria [17]: 0, no neurological deficits; 1, unable to extend the contralateral forelimb; 2, circling to the paretic side; 3, falling to the contralateral side; and 4, unable to engage in spontaneous activity.

### 2.5. Infarct Volume Measurement

To determine the effect of geraniin on brain infarction, intact brains were quickly removed after neurological deficit evaluation. The brains were cut into five 2 mm thick coronal sections and incubated for 30 min in 2% TTC solution at 37°C. All images were collected and analyzed using ImageJ (NIH, USA) [18].

### 2.6. Hematoxylin and Eosin (H&E) Staining

The rats were anesthetized 72 h after MCAO/R and perfused with 4% paraformaldehyde. Next, the brains were rapidly removed and fixed with 4% paraformaldehyde overnight. The brains were then embedded in paraffin and cut into 6 *μ*m thick sections. Subsequently, the sections were deparaffinized in xylene and rehydrated through graded alcohol solutions. Then, the sections were stained with HE. Morphological changes in cerebral cortex and hippocampal tissues were observed under a light microscope (Olympus, Tokyo, Japan).

### 2.7. Terminal Deoxynucleotidyl Transferase- (TdT-) Mediated d-UTP Nick End Labeling (TUNEL) Staining

TUNEL staining was performed to determine the cell apoptosis rate as previously described [19]. Brains were sliced into 4 *μ*m thick frozen sections. The number of TUNEL-positive cells in the cerebral cortex and hippocampus was counted in four randomly selected rats from each group. The number of TUNEL-positive cells was determined by an observer blinded to the study design. Images were collected using an automatic fluorescence microscope and analyzed using ImageJ software (NIH, USA).

### 2.8. Oxidative Stress Measurement

#### 2.8.1. SOD Activity

SOD activity in the serum, brain tissues, and PC12 cells was measured according to the instructions of the kit. Substrate application solution and enzyme working solution were prepared, and the samples to be tested were added to these solutions and incubated at 37°C for 20 min. An enzyme immunoassay reader was used to measure the absorbance at 450 nm. SOD activity (U/L) was calculated as the SOD inhibition rate/50% × 2/the concentration of the tested sample.

#### 2.8.2. LDH Activity

Working solution was prepared according to the instructions of the kit, and the sample to be tested was added and incubated at room temperature for 5 min. An enzyme immunoassay reader was used to measure the absorbance at 450 nm. LDH activity (U/L) was calculated as (test OD–control OD)/(standard OD–negative control OD) × the concentration of the standard (0.2 nmol/mL) × 1000.

#### 2.8.3. MDA Content

Working solution was prepared according to the instructions of the kit, and the samples to be tested were added and incubated in water at 95°C for 1 h. After removal, the samples were cooled with running water and centrifuged at 4000 r/min for 10 min. The supernatant was collected for analysis. An enzyme immunoassay reader was used to measure the absorbance at 532 nm. MDA content was calculated as (test OD–control OD)/(standard OD–negative control OD) × the concentration of the standard (10 nmol/mL).

#### 2.8.4. NO Content

Working solution was prepared according to the instructions of the kit, and the samples to be tested were added and incubated at room temperature for 15 min. An enzyme immunoassay reader was used to measure the absorbance at 550 nm. NO content (*μ*mol/L) was calculated as (test OD–control OD)/(standard OD–negative control OD) × 20 *μ*mol/L × 2/the concentration of the tested sample.

#### 2.8.5. nNOS Content

Working solution was prepared according to the instructions of the kit, and the samples to be tested were added and incubated at 37°C for 30 min. An enzyme immunoassay reader was used to measure the absorbance at 450 nm. According to the kit instructions, ELISA calc software was used for calculation, and a logistic curve was selected for fitting the model.

### 2.9. Cell Culture and Treatment

PC12 cells were purchased from the Kunming Institute of Zoology, Chinese Academy of Science. They were cultured in DMEM1640 supplemented with 10% FBS and 1% penicillin-streptomycin and incubated at 37°C in 5% CO_2_ and 95% O_2_. To mimic the cerebral I/R injury, an in vitro cell model of OGD/R was established as previously described [20]. First, PC12 cells incubated with glucose-free medium were placed in an incubator at 37°C, 1% O_2_, 95% N_2_, and 5% CO_2_ (SHel Lab, China). Two hours after oxygen-glucose deprivation (OGD), the PC12 cells were placed in a normal incubator, and the glucose-free medium was removed. The medium of the cells in the OGD/R model group was replaced with fresh complete medium, while that of the drug administration group was replaced with medium containing geraniin at different concentrations (0.1 *μ*mol/L, 1 *μ*mol/L, and 10 *μ*mol/L) or nimodipine (10 *μ*mol/L). After 24 h of incubation, the cells were collected for subsequent experiments.

### 2.10. Flow Cytometric Analysis

The cell apoptosis rate was measured after OGD/R by flow cytometry according to the manufacturer's protocol [21]. Briefly, the cells were washed twice with ice-cold PBS and stained with FITC-conjugated Annexin V and propidium iodide (PI) at room temperature for 15 min in the dark using an Annexin V-FITC Apoptosis Detection Kit. The stained cells were analyzed using flow cytometry as soon as possible (within 1 h). Annexin V-FITC+/PI− and Annexin V-FITC+/PI+ cells were considered early apoptotic and late apoptotic cells, respectively. The apoptotic rate was calculated as the percentage of early/primary apoptotic cells (Annexin V+/PI) and late/secondary apoptotic cells (Annexin V+/PI+) [22].

### 2.11. Western Blotting

Proteins were extracted from brain tissues of the ischemic side and PC12 cells, separated on 10%–15% SDS-PAGE gels, and transferred to membranes. Then, the membranes were incubated with primary antibodies against Bax (1 : 1000, ABclonal, China), Bcl-2 (1 : 1000, ABclonal, China), caspase-3 (1 : 1000, ABclonal, China), cleaved caspase-3 (1 : 1000, ABclonal, China), Nrf2 (1 : 1000, Cell Signaling Technology, USA), HO-1 (1 : 1000, Proteintech, USA), Lamin B (1 : 1000, Abcam, USA), GAPDH (1 : 1000, Proteintech, USA), and *β*-actin (1 : 1000, Proteintech, USA). After three washes with TBST, the membranes were incubated with corresponding secondary antibodies (1 : 6500, Abcam, USA) and scanned using a BIO-RAD imaging system (BIO-RAD Gel Doc XR, USA). The band intensity was normalized to the intensity of the GAPDH or Lamin B band and analyzed using Image Lab v5.2 software.

### 2.12. Statistical Analysis

The data are expressed as the means ± SEM. One-way analysis of variance (ANOVA) was used to analyze the significance of differences between groups. *P* values below 0.05 were considered significant.

## 3. Results

### 3.1. Geraniin Exerts a Protective Effect against the I/R Injury In Vivo

**Figure 1:**
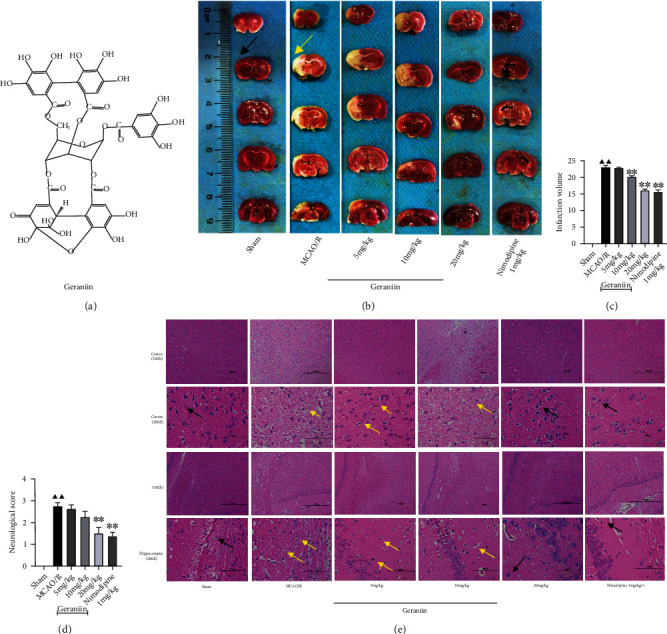
The effect of geraniin on the infarct volume, neurological scores, and neuronal morphology following 72 h of reperfusion after 2 h of MCAO. (a) The chemical structure of geraniin. (b) Representative brain sections (2 mm thick) stained with TTC after 72 h of MCAO and treatment with 5, 10, and 20 mg/kg geraniin and 1 mg/kg nimodipine. Normal brain tissue is red and is indicated by the black arrow, while infarcted tissue is pale gray and is indicated by the yellow arrow. (c) Quantitative analysis of the infarct size in each group. (d) Quantitative analysis of neurological scores in each group at 72 h after reperfusion. (e) Pathological changes in the cerebral cortex and hippocampus were detected by HE staining (100x, scale bar = 500 *μ*m; 200x, scale bar = 100 *μ*m). The black arrows indicate intact nerve cells, and the white arrows indicate damaged nerve cells. The data are expressed as the means ± SEM (*n* = 8). ^▲▲^*P* < 0.01 vs. the sham group; ^∗^*P* < 0.05, ^∗∗^*P* < 0.01 vs. the MCAO/R group.

As shown in Figures 1(b)–1(d), the infarct volume and neurological scores were increased in the MCAO/R group compared to the sham group (*P* < 0.01). In contrast, geraniin (10 and 20 mg/kg) and nimodipine (1 mg/kg) treatment significantly decreased the infarct volume and neurological scores (*P* < 0.01). We also conducted HE staining to observe neuronal morphology in the cerebral cortex and hippocampus. As revealed in Figure 1(e), most neurons in the sham group appeared round, had large nuclei, and were arranged in a regular pattern. In the MCAO/R group, the injured neurons were disarranged and shrunken and had dark nuclei. However, the number of surviving neurons was significantly increased in the geraniin- (20 mg/kg) and nimodipine- (1 mg/kg) treated groups compared with the MCAO/R group. These results indicated that geraniin protects against I/R injury in rats in vivo.

### 3.2. Geraniin Suppresses MCAO/R-Induced Oxidative Stress In Vivo

**Figure 2:**
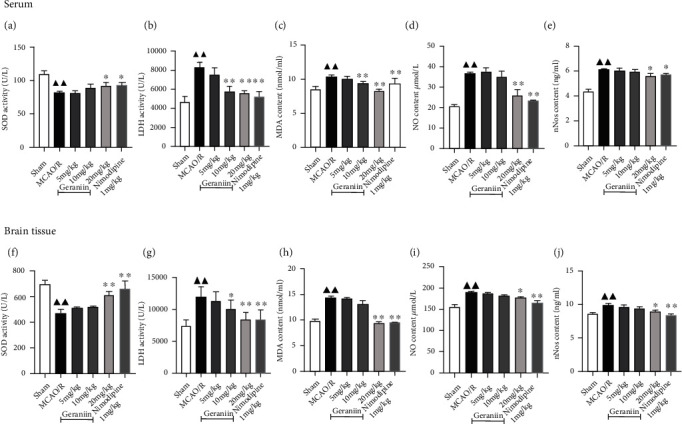
Effect of geraniin on oxidative stress 72 h after MCAO in rats. (a–e) Effects of geraniin on serum SOD and LDH activity and MDA, NO, and nNOS contents. (f–j) Effects of geraniin on SOD and LDH activity and MDA, NO, and nNOS contents in brain homogenates from rats. The data are expressed as the means ± SEM (*n* = 8). ^▲▲^*P* < 0.01 vs. the sham group; ^∗^*P* < 0.05, ^∗∗^*P* < 0.01 vs. the MCAO/R group.

Next, we evaluated the effect of geraniin against oxidative stress in a rat model of MCAO/R. As shown in Figure 2, the activity of SOD in the sera and brain tissues in the MCAO/R group was lower than that in the sham group (*P* < 0.01). Compared with that in the MCAO/R group, the level of SOD in the geraniin- (20 mg/kg) and nimodipine- (1 mg/kg) treated groups was significantly higher (*P* < 0.05 or *P* < 0.01). The levels of LDH, MDA, NO, and nNOS in the MCAO/R group were significantly increased compared with those in the sham group (*P* < 0.01). Geraniin significantly decreased the levels of these molecules in a dose-dependent manner (*P* < 0.05 and *P* < 0.01). These results suggest that geraniin treatment can obviously suppress oxidative stress induced by MCAO/R.

### 3.3. Geraniin Attenuates MCAO/R-Induced Neuronal Apoptosis In Vivo

**Figure 3:**
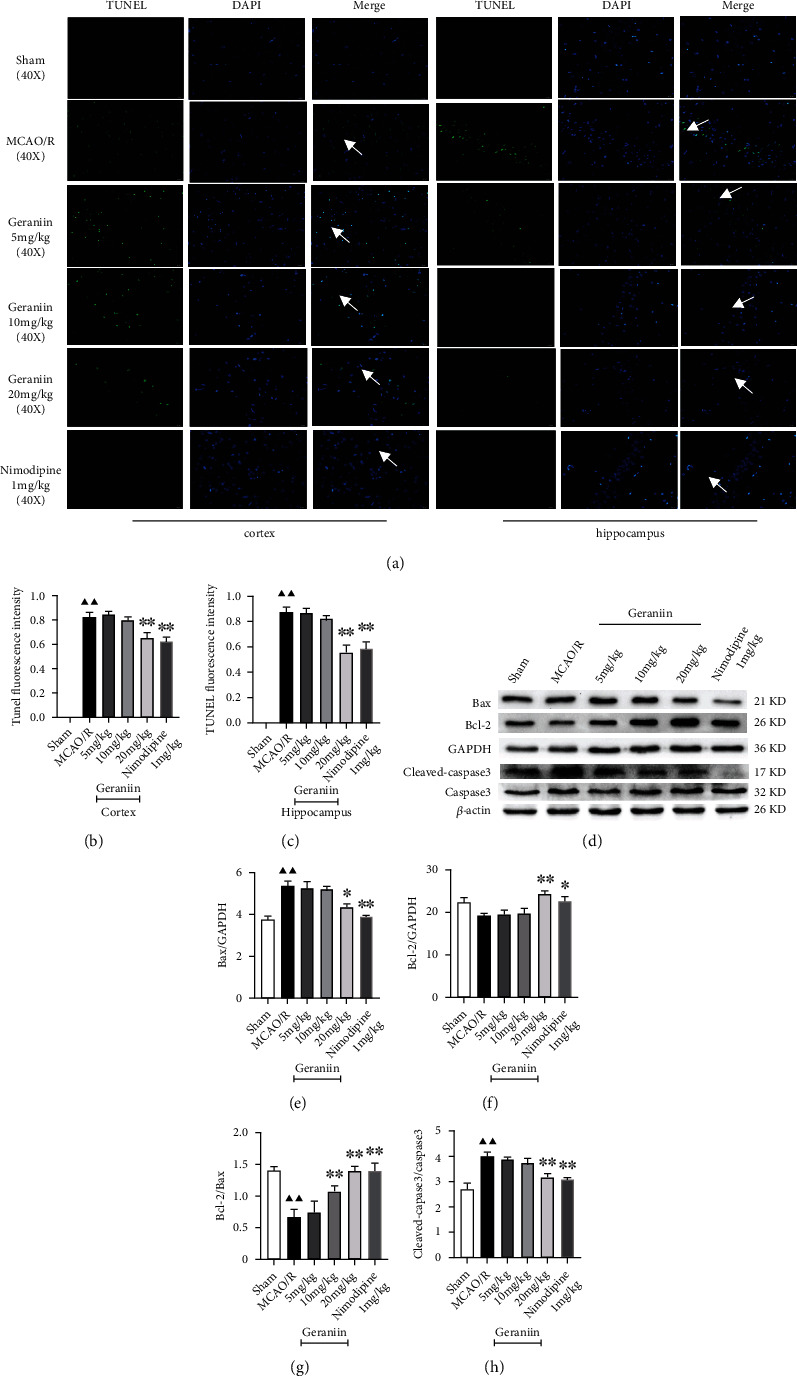
The effect of geraniin on neuronal apoptosis 72 h after MCAO in rats. (a–c) Effect of geraniin on MCAO/R-induced neuronal apoptosis in cerebral cortex and hippocampal sections, as determined by TUNEL staining (40x, scale bar = 20 *μ*m) (*n* = 4). The white arrows indicate TUNEL-positive cells. (d–h) The expression levels of Bax, Bcl-2, cleaved caspase-3, and caspase-3 were measured by Western blot analysis (*n* = 6). The data are expressed as the means ± SEM. ^▲▲^*P* < 0.01 vs. the sham group; ^∗^*P* < 0.05, ^∗∗^*P* < 0.01 vs. the MCAO/R group.

To investigate the protective effects of geraniin against MCAO/R-induced neuronal apoptosis, TUNEL staining and Western blotting were performed. As shown in Figure 3, few TUNEL-positive cells were present in cerebral cortex and hippocampal sections from the sham group. The number of TUNEL-positive cells was more significantly increased in the MCAO/R group than in the sham group (*P* < 0.01). Treatment with geraniin (20 mg/kg) and nimodipine (1 mg/kg) significantly decreased the number of TUNEL-positive cells (*P* < 0.05 or *P* < 0.01). In addition, compared with that in the sham group, the expression of Bax and cleaved caspase-3 in the MCAO/R group was significantly enhanced, whereas the expression of Bcl-2 was significantly reduced, resulting in a low Bcl-2/Bax expression ratio (*P* < 0.05 or *P* < 0.01). Geraniin decreased the expression of Bax and cleaved caspase-3 but significantly increased the expression of Bcl-2 in a dose-dependent manner, resulting in a high Bcl-2/Bax expression ratio (*P* < 0.05 or *P* < 0.01). These results suggest that geraniin treatment can attenuate MCAO/R-induced neuronal apoptosis in vivo.

### 3.4. Geraniin Protects PC12 Cells from OGD/R-Induced Cytotoxicity and Oxidative Stress

**Figure 4:**
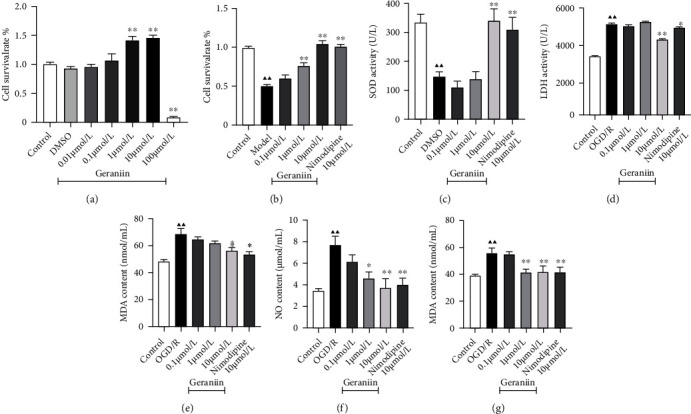
The effect of geraniin on cell viability and oxidative stress in PC12 cells after OGD/R. (a) The survival rate of geraniin-treated PC12 cells. (b) Effect of geraniin on the viability of PC12 cells subjected to OGD/R. (c–g) Effects of geraniin on SOD and LDH activity and MDA, NO, and nNOS contents in PC12 cells subjected to OGD/R. The data are expressed as the means ± SEM (*n* = 8). ^▲▲^*P* < 0.01 vs. the control group; ^∗^*P* < 0.05, ^∗∗^*P* < 0.01 vs. the OGD/R group.

To verify the safe concentration range of geraniin in PC12 cells, we treated PC12 cells with different concentrations of geraniin (0.01, 0.1, 1, 10, and 100 *μ*mol/L) for 24 h. As presented in Figure 4(a), the cell survival rate of the geraniin- (100 *μ*mol/L) treated group was significantly decreased compared with that of the control group (*P* < 0.01). Thus, we used a concentration of 0.1–10 *μ*mol/L for subsequent studies of the effect of geraniin. OGD/R significantly decreased the cell survival rate (*P* < 0.01), indicating cell death. Compared with that of OGD/R-treated cells, the survival rate of the OGD/R-treated PC12 cells administered with geraniin (1 and 10 *μ*mol/L) was significantly increased in a concentration-dependent manner (*P* < 0.01). Additionally, the level of SOD was significantly lower in the OGD/R group than in the control group (*P* < 0.01). Compared with that in the OGD/R group, the level of SOD in the geraniin- (10 *μ*mol/L) and nimodipine- (10 *μ*mol/L) treated groups was significantly higher (*P* < 0.01). As shown in Figures 4(d)–4(g), the levels of LDH, MDA, NO, and nNOS in the OGD/R group were significantly increased compared with those in the control group (*P* < 0.01). In contrast, geraniin reduced the levels of LDH, MDA, NO, and nNOS in a concentration-dependent manner (*P* < 0.05 or *P* < 0.01). These results indicate that geraniin can protect PC12 cells from OGD/R-induced cytotoxicity and oxidative stress.

### 3.5. Geraniin Protects PC12 Cells from OGD/R-Induced Cell Apoptosis

**Figure 5:**
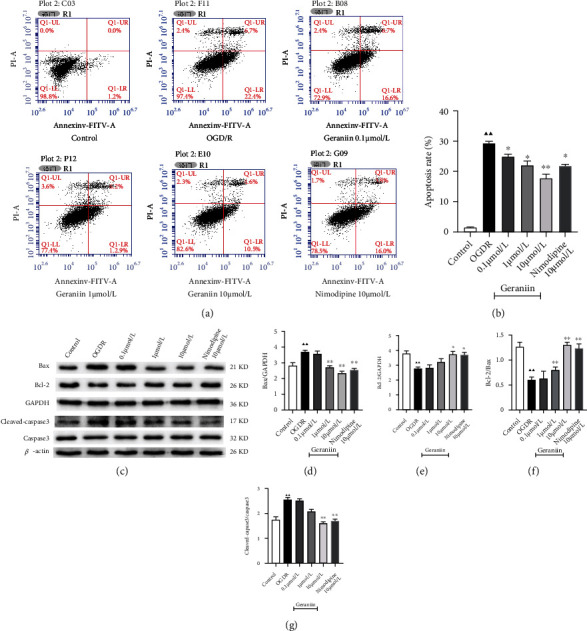
The effect of geraniin on OGD/R-induced apoptosis of PC12 cells. (a, b) Effect of geraniin on the OGD/R-induced cell apoptosis rate, as determined by flow cytometry. (c–f) The protein expression of Bax, Bcl-2, caspase-3, and cleaved caspase-3 was measured by Western blot analysis. The data are expressed as the means ± SEM (*n* = 8). ^▲▲^*P* < 0.01 vs. the control group; ^∗^*P* < 0.05, ^∗∗^*P* < 0.01 vs. the OGD/R group.

We also assessed the effect of geraniin on OGD/R-induced apoptosis of PC12 cells by flow cytometry and Western blot analysis. As shown in Figures 5(a) and 5(b), the apoptosis rate of PC12 cells increased from 1.2% to 29.1% after exposure to OGD/R (*P* < 0.01), whereas treatment with geraniin (0.1, 1, and 10 *μ*mol/L) significantly decreased the cell apoptosis rate after OGD/R in a concentration-dependent manner (*P* < 0.05 or *P* < 0.01). Moreover, as presented in Figures 5(c)–5(g), the expression of apoptosis-related proteins, such as Bax and cleaved caspase-3, was enhanced and Bcl-2 expression was decreased in PC12 cells exposed to OGD/R (*P* < 0.01). Treatment with geraniin (10 *μ*mol/L) and nimodipine (10 *μ*mol/L) markedly reduced the protein expression of Bax and cleaved caspase-3 and elevated Bcl-2 expression, resulting in a significant increase in the Bcl-2/Bax ratio. These results demonstrate that geraniin can protect PC12 cells from OGD/R-induced cell apoptosis.

### 3.6. Geraniin Regulates the Nrf2/HO-1 Signaling Pathway In Vivo and In Vitro

**Figure 6:**
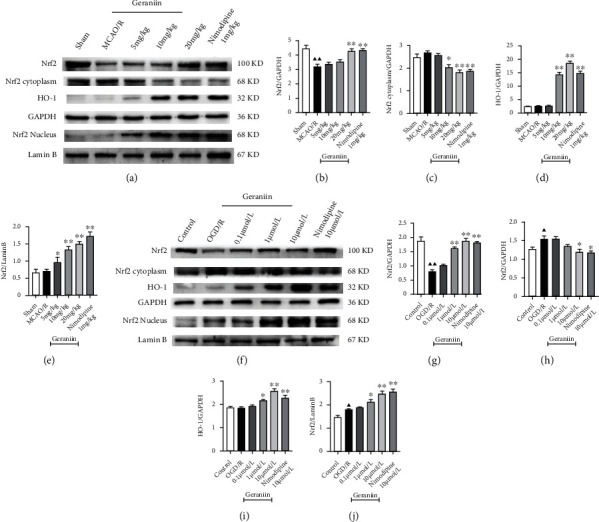
The effect of geraniin on the Nrf2/HO-1 signaling pathway in vivo and in vitro. (a–e) Protein expression levels of total Nrf2, cytoplasmic Nrf2, nuclear Nrf2, and HO-1 in the MCAO rat model after 72 h of reperfusion. (f–j) Protein expression levels of total Nrf2, cytoplasmic Nrf2, nuclear Nrf2, and HO-1 in PC12 cells exposed to OGD/R. The data are expressed as the means ± SEM (*n* = 8). ^▲▲^*P* < 0.01 vs. the sham or control group; ^∗^*P* < 0.05, ^∗∗^*P* < 0.01 vs. the MCAO/R or OGD/R group.

To evaluate whether the neuroprotective effect of geraniin is related to the Nrf2/HO-1 signaling pathway, the protein expression of total Nrf2, cytoplasmic Nrf2, nuclear Nrf2, and HO-1 was evaluated by Western blotting. As shown in Figures 6(a)–6(e), compared with that in the sham group, total Nrf2 protein expression in the MCAO/R group was significantly decreased, while the expression of nuclear Nrf2 was increased (*P* < 0.01). However, there was no significant difference in the expression of cytoplasmic Nrf2 and HO-1 between these two groups. Treatment with geraniin (10 and 20 mg/kg) and nimodipine (1 mg/kg) markedly decreased the expression of Nrf2 in the cytoplasm in a concentration-dependent manner and increased total Nrf2, nuclear Nrf2, and HO-1 protein expression (*P* < 0.05 or *P* < 0.01). Consistent with the in vivo data, the expression of total Nrf2, cytoplasmic Nrf2, nuclear Nrf2, and HO-1 showed similar changes in the geraniin-treated group in vitro. These results indicated that geraniin can facilitate the activation of the Nrf2/HO-1 signaling pathway in vivo and in vitro.

## 4. Discussion

In this study, we confirmed for the first time that geraniin exerts a neuroprotective effect against cerebral I/R injury. Our study demonstrated that treatment with geraniin ameliorated cerebral I/R injury by decreasing the infarct volume and improving neurological scores. Moreover, geraniin administration attenuated oxidative stress and neuronal apoptosis in vivo and in vitro. Furthermore, the neuroprotective effect of geraniin was associated with activation of the Nrf2/HO-1 pathway.

The protective effect of geraniin, as a natural polyphenol, has attracted the attention of a few researchers. Youn and Jun found that geraniin alleviates oxidative damage and neuroinflammation in A*β*_25–35_-treated PC12 cells [23]. Wang et al. reported that geraniin mitigates LPS-elicited neural/synaptic neurodegeneration, amyloidogenesis, neuroinflammation, and cognitive impairment [24]. However, the protective effect and molecular mechanism of geraniin in ischemic stroke and cerebral I/R injury have not been reported. Our results showed that treatment with geraniin significantly decreased the infarct volume, improved neurological scores, ameliorated pathological changes in neurons in the cortex and hippocampus after MCAO/R, and increased the survival rate of OGD/R-treated PC12 cells. These results suggest that geraniin exerts a protective effect against cerebral I/R injury.

After cerebral I/R injury, oxygen free radicals are crucial protagonists of oxidative stress and cause neuronal injury and death [25]. Multiple antioxidants have been shown to ameliorate cerebral I/R injury, strongly indicating that suppression of oxidative stress is an attractive potential therapeutic target for counteracting cerebral I/R injury [26]. During cerebral I/R injury, oxygen free radicals are produced, SOD activity is decreased, and LDH activity and MDA content are increased, leading to lipid peroxidation, which further impairs nerve cell function and results in brain damage. At this time, nNOS, the level of which is increased, catalyzes the synthesis of NO, which aggravates neuronal injury [27]. Studies have found that treatment with geraniin reduces the increase in MDA content and significantly decreases SOD activity, thereby alleviating obesity and its pathophysiological sequelae by reducing oxidative stress [28]. However, no studies have shown that geraniin can reduce oxidative stress induced by cerebral I/R. In our study, we found that geraniin (20 mg/kg) significantly increased SOD activity, decreased LDH activity, and reduced MDA, NO, and nNOS contents in the sera and brain tissues of rats subjected to MCAO/R. The in vitro results were consistent with the in vivo findings. These results directly show that geraniin attenuates the cerebral I/R injury by suppressing oxidative stress.

Oxidative stress and apoptosis are involved in the pathological process of cerebral I/R injury. Inhibition of oxidative stress and apoptosis by drug intervention can benefit the treatment of cerebral I/R injury [29]. Accumulating evidence indicates that cerebral I/R injury can cause the production and release of excessive free radicals and reactive oxygen species, which mediate the apoptosis pathway in neurons [30]. Neuronal apoptosis plays a key role in the cerebral I/R injury [31]. Apoptosis is promoted by activation of the caspase family of cysteine proteases, which is one mechanism of programmed cell death regulation [32]. During the process of cerebral I/R injury, apoptosis is most closely related to the antiapoptotic protein Bcl-2 and the proapoptotic protein Bax, which are members of the Bcl-2 family of proteins. Additionally, whether apoptosis occurs is determined by the Bcl-2/Bax expression ratio, and a decrease in the Bcl-2/Bax ratio has been reported to be related to an increase in apoptosis [33]. Furthermore, activation of caspase-3 leads to the occurrence of apoptotic events [34]. Studies have revealed that geraniin exerts significant inhibitory effects on tumor growth and markedly promotes cancer cell apoptosis by increasing the expression of Bax, caspase-3, and caspase-9 and decreasing the level of Bcl-2 [35]. However, no studies have demonstrated that geraniin can play a neuroprotective role by exerting antiapoptotic effects in cerebral I/R injury. In the present study, geraniin obviously reduced the number of TUNEL-positive cells in vivo and decreased the rate of apoptosis in vitro. Moreover, in vivo and in vitro experiments indicated that treatment with geraniin significantly increased the expression level of Bcl-2 and decreased the expression levels of cleaved caspase-3 and Bax, thus increasing the Bcl-2/Bax ratio. The above results indicate that geraniin can play a neuroprotective role by affecting the expression of apoptosis-related proteins.

Nrf2 is one of the important transcription factors in the endogenous defense system. The Nrf2-mediated signaling pathway can alleviate cerebral I/R injury through antioxidative stress, antiapoptosis, anti-inflammation, and other ways; it has been used as a key target for the treatment of ischemic stroke [36]. Studies have shown that after the cerebral I/R injury, oxygen free radicals can trigger Nrf2 phosphorylation and nuclear translocation, initiate the transcription of the downstream target gene HO-1, and induce the production of a variety of endogenous antioxidant enzymes, thereby reducing or eliminating the production of oxygen free radicals, resulting in redox balance in the body [37, 38]. Studies have found that in both the peri-infarct and core infarct regions, Nrf2 expression begins to increase at 2 h, peaks at 8 h, and then decreases at 24 and 72 h of reperfusion in a mouse transient middle cerebral artery (tMCAO) model [39]. These results suggest that activation of the Nrf2/HO-1 pathway is associated with alleviating oxidative stress-induced damage. Meanwhile, the Nrf2/HO-1 signaling pathway is also involved in the regulation of apoptosis. Some studies confirmed that when the Nrf2 nuclear protein was inhibited in the MCAO/R rat model, the expression of the proapoptotic protein Bax increased and the expression of the antiapoptotic protein Bcl-2 decreased, thereby increasing the apoptosis rate [40, 41]. Furthermore, inhibition of the Nrf2/HO-1 signaling pathway also can aggravate oxidative stress and apoptosis. Shih et al. [42] found that compared with wild-type mice, Nrf2-knockout mice treated with tMCAO exhibit decreased antioxidant enzyme activity, increased apoptosis, raised cerebral infarction area, and aggravated behavioral deficits and cerebral I/R injury. Thus, the Nrf2/HO-1 signaling pathway plays an important role in antioxidant stress and apoptosis. In the present study, we found that geraniin significantly decreased the expression of Nrf2 in the cytoplasm in a concentration-dependent manner and increased total Nrf2, nuclear Nrf2, and HO-1 protein expression in vivo and in vitro. These results indicate that the neuroprotective mechanism of geraniin may be related to the activation of the Nrf2/HO-1 signaling pathway.

## 5. Conclusion

This study reports for the first time that geraniin exerts a neuroprotective effect against cerebral I/R injury both in vivo and in vitro, as indicated by improvements in neurological deficits, a reduction in the infarct volume, and decreases in oxidative stress and neuronal apoptosis, via regulation of the Nrf2/HO-1 signaling pathway. More experiments are needed in the future to reveal and elucidate the possible mechanisms underlying the neuroprotective effects of geraniin.

## Data Availability

The data used to support the findings are available from the corresponding author upon request.

## Abbreviations

CIRI: Cerebral ischemia/reperfusion injury
MCAO/R: Middle cerebral artery occlusion/reperfusion
OGD/R: Oxygen glucose deprivation/reoxygenation
TTC: 2,3,5-Triphenyltetrazolium chloride
HE: Hematoxylin and eosin
SOD: Superoxide dismutase
LDH: Lactate dehydrogenase
MDA: Malondialdehyde
NO: Nitric oxide
nNOS: Neuronal nitric oxide synthase
Nrf2: Nuclear factor E2-related factor 2
HO-1: Heme oxygenase-1.
